# Reply to “Checking the shape of hepatocellular carcinoma: How irregular is irregular?”

**DOI:** 10.1007/s00535-026-02394-5

**Published:** 2026-03-21

**Authors:** Feng Zhang, Yong-Shuai Wang, Lian-Xin Liu, Ji-Zhou Wang

**Affiliations:** 1 Department of Hepatobiliary Surgery, Centre for Leading Medicine and Advanced Technologies of IHM, The First Affiliated Hospital of USTC, Division of Life Sciences and Medicine, University of Science and Technology of China, Hefei, Anhui 230001 China; 2 Anhui Province Key Laboratory of Hepatopancreatobiliary Surgery, Hefei, Anhui 230001 China; 3 Anhui Provincial Clinical Research Center for Hepatobiliary Diseases, Hefei, Anhui 230001 China

**Keywords:** Immune checkpoint inhibitor, Prognosis, Hepatocellular carcinoma, Tumor shape, Tumor burden

We greatly appreciate the insightful and constructive comments by Professors Huo and Ho on our study establishing the alpha-fetoprotein (AFP) and tumor shape irregularity (ATSI) score for survival prediction in advanced hepatocellular carcinoma (HCC) patients receiving immune checkpoint inhibitors (ICIs) [1]. The pioneering work of Professor Huo and colleagues in HCC prognostic stratification has provided valuable guidance for both clinical practice and our research, and we are pleased to respond to the specific points raised in this letter.

In our original study, tumor shape irregularity was defined using four explicit CT/MRI criteria. To minimize interobserver bias, these criteria were applied independently by three senior radiologists under blinded conditions. The prognostic significance of this definition was subsequently validated in a randomized cohort. More recently, we conducted a quantitative analysis of tumor morphology using AI-based radiomics with three-dimensional (3D) stereoscopic liver tumor modeling. The results show that numerically defined 3D shape irregularity is significantly associated with poor prognosis in HCC, corroborating our initial conclusions.

We acknowledge the well-validated prognostic value of the tumor burden score (TBS) in HCC risk stratification [2], and note with interest that the TBS-ALBI system from Professor Huo’s team further expands its clinical utility, with consistent prognostic performance across diverse HCC etiologies, BCLC stages, and treatment modalities [3]. Notably, the Taipei Integrated Scoring System developed by Professor Huo and colleagues was the first to adopt total tumor volume (TTV) in constructing an HCC prognostic model. This approach aligns with our view that tumor volume reflects real tumor burden more accurately than the diameter‑ and number-based parameters used in TBS [4]. In addition, we examined the ratio of total tumor volume to total liver volume as a means to account for individual differences. Our preliminary propensity score matched analysis suggests that this ratio is an independent predictor of HCC survival, with patients in the high tumor burden group showing significantly worse outcomes.

In conclusion, our ongoing investigation suggests that both tumor shape and tumor burden are important prognostic determinants in HCC. The ATSI score, derived from routine AFP levels and tumor shape, offers a simple and practical tool for risk stratification in advanced HCC patients receiving ICIs, supporting personalized treatment decisions. Its broader clinical utility requires confirmation through large-scale prospective studies.
